# Glutathione Transferases Omega-1 and -2 Polymorphisms in Cancer: Drivers or Silent Bystanders?

**DOI:** 10.3390/ijms26146586

**Published:** 2025-07-09

**Authors:** Eugenia Belcastro, Giulia Paties Montagner, Alfonso Pompella, Simona Piaggi, Alessandro Corti

**Affiliations:** Department of Translational Research NTMS, University of Pisa Medical School, via Roma, 55, 56126 Pisa, Italy; eugenia.belcastro@unipi.it (E.B.); g.patiesmontagner@studenti.unipi.it (G.P.M.); alfonso.pompella@unipi.it (A.P.); alessandro.corti@unipi.it (A.C.)

**Keywords:** glutathione transferases omega, cancer, polymorphisms, gene variant, genetic susceptibility, risk factor

## Abstract

The omega class of glutathione transferases (GSTOs) includes two enzymes that catalyze atypical reactions, influencing key cellular processes such as cell survival, proliferation, drug resistance, and inflammation. In recent years, numerous studies have focused on GSTOs’ role and on the significance of their polymorphisms in cancer risk and progression; though findings have been somewhat inconsistent. This systematic review aims to critically evaluate the current literature to determine whether GSTOs’ polymorphisms may represent significant contributors to tumor progression, by analyzing their association with severity, mortality, and disease progression across different cancer types. Although for some types of neoplasms the studies reporting positive correlations are the majority, the role of GSTOs’ polymorphisms in cancer remains inconclusive due to conflicting findings, limited data on rare variants, and multiple confounding factors; further research is needed to clarify their tissue-specific and context-dependent effects.

## 1. Introduction

The glutathione-transferase (GST) superfamily consists of a group of isoenzymes involved in the phase II detoxification of endogenous as well as exogenous compounds, including drugs, pesticides, and other environmental pollutants [1,2].

GSTs are expressed ubiquitously in Prokaryotes and Eukaryotes. In Mammals, three major protein families have been identified, based on their subcellular localization. The cytosolic GSTs represent the largest group with at least seven classes of isoenzymes: alpha (A), mu (M), omega (O), pi (P), sigma (S), theta (T), and zeta (Z). The kappa (K) class is expressed in the mitochondria and peroxisomes, whereas the membrane-bound MAPEG (Membrane-Associated Proteins in Eicosanoid and Glutathione Metabolism) enzymes have a microsomal localization [3,4].

The enzymes belonging to the omega class (GSTO) are characterized by a catalytically active cysteine residue (Cys32) in their active site [5], replacing the tyrosine or the serine residues expressed by other GSTs [6]. This structural feature has relevant functional effects on the type of reactions catalyzed by GSTOs.

In recent years, numerous studies focused on the significance of GSTOs’ functions and polymorphisms in cancer risk and progression.

While the reactions catalyzed by GSTOs present interesting perspectives for the process of carcinogenesis, the implications of its polymorphisms are less clear. The undoubted biological significance of GSTOs’ functions, identified early but not yet fully explored, justifies the active research in this field, both from a biochemical and molecular point of view. However, the supposed correlations between GSTOs’ polymorphisms and the risk or progression of certain types of cancer have not been confirmed in all the studies, and the picture seems to be more complicated than expected.

The biological role of GSTOs in specific tissues, the overlapping enzymatic functions, and the heterogeneous sample size may contribute to the variability in the available data. This complexity has indeed led recent studies to focus on haplotypes, rather than single GSTOs’ polymorphisms—including GSTOs’ variants as well, when assessing associations with cancer risk or progression.

This review aims to critically evaluate the current data available in the literature on this specific topic. This manuscript is structured in two main parts. The first part (paragraphs 2 and 3) summarizes established findings on GSTOs’ substrate specificity and polymorphisms, thus introducing and highlighting the key points for the subsequent discussion. The second part (paragraph 4) presents a systematic review of studies examining GSTOs’ polymorphisms in cancer, which is organized by cancer type to ensure a clearer and more organic discussion.

## 2. The Omega Class of GSTs

To date, two functional omega class GST genes are known in humans (*GSTO1*, *GSTO2*), both located on chromosome 10q24.3. A pseudogene, *GSTO3p*, has also been identified on chromosome 3 [7].

The *GSTO1* gene contains six exons and spans 12.5 kb, whereas the *GSTO2* gene is localized approximately 7.5 kb downstream of *GSTO1* and is composed of six coding exons and spans 24.5 kb [7]. The human *GSTO1* cDNA encodes a protein of 241 amino acids with a 64% identity to the 243 residues of GSTO2, and both proteins have a conserved cysteine residue at position 32 (Cys32) in the active site [5,7].

GSTO1-1 is ubiquitously expressed in human tissues with the greatest expression in the liver, skeletal muscle, and heart [5]. The expression of GSTO2-2 is less widespread, with relatively high levels of expression in the liver, kidney, and skeletal muscle but the highest expression is in the testis [7,8].

The structures of recombinant human GSTO1-1 and GSTO2-2 have been determined by X-ray crystallography [5,9,10,11] and provided insights into the structure of their active site. In particular, GSTO1-1 presents with a relatively open active site pocket, lined with some polar residues, that can potentially harbor proteins or peptides as substrates [5].

**Figure 1:**
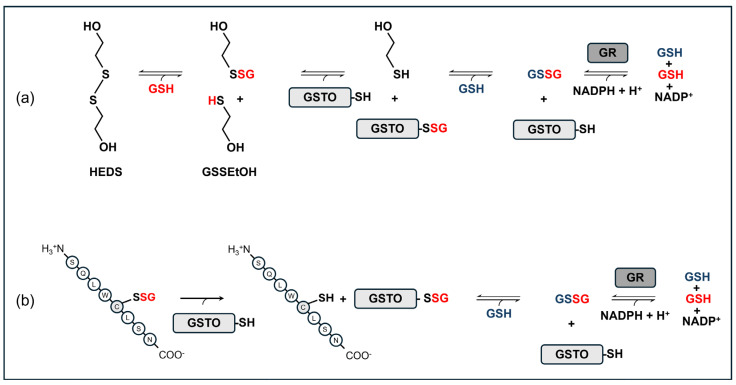
Schematic representation of assays used to measure thioltransferase (**a**) and deglutathionylation (**b**) activities of GSTOs.

GST typically metabolizes substrates by conjugating them with glutathione (GSH), i.e., by catalyzing the nucleophilic attack of the thiol of GSH at the electrophilic center of the drug or metabolite. Nevertheless, early studies promptly demonstrated that GSTO1-1 and GSTO2-2 are essentially inactive against the classical substrates processed by other GSTs [5] and rather catalyze the reduction of different substrates such as dehydroascorbate (DHA; [11,12,13]), arsenate, methylarsonate (MMA^V^), and dimethylarsinate (DMA^V^) [12,14,15] (Table 1). Moreover, GSTO1-1 can also catalyze glutaredoxin (Grx)-like thioltransferase reactions with a substrate such as 2-hydroxyethyldisulfide (Figure 1a; [16]) and the more specific biotransformation of α-haloketones [17]. A critical role of Cys32 in the active site of GSTOs was confirmed by studies demonstrating that its substitution with an Ala residue (C32A) can totally prevent the thioltransferase activity and the biotransformation of α-haloketones. Indeed, Cys32 forms a disulfide bond with glutathione as part of the catalytic mechanism in the thiol transferase reaction [16]. On the other hand, the C32A enzyme had an increased dehydroascorbate reductase activity, suggesting that Cys32 is not required for the reduction of DHA [18].

Based on the structural characteristics of the active site, further studies focused on the possibility that GSTOs could be able to catalyze glutathionylation reactions as well. Indeed, GSTO1-1, but not *GSTO2-2*, was capable of deglutathionylating a model of synthetic glutathionylated peptide (Figure 1b; [18]), i.e., the reduction of the mixed disulphide between glutathione and the model peptide. This finding was further supported by experiments on T47-D breast cancer cells where the expression of recombinant GSTO1-1 caused a significant reduction in the level of intracellular protein glutathionylation [18]. This activity could have important functional implications, mainly depending on GSTO1-1 substrates. S-thiolation has emerged as a major post-translational modification of proteins, both protecting them from irreversible oxidation and possibly modulating their functions. S-thiolation/de-thiolation reactions—thanks to their reversibility and fast turnover rate—can also act as a redox signal pathway to turn on and off the transcription of genes [19,20].

To date, a limited number of GSTO1-1 substrates for deglutathionylation have been identified [21]. β-Actin, heat shock protein 70, heat shock protein 7C, and the prolactin-inducible protein have been demonstrated to be specific targets for GSTO1-1-mediated deglutathionylation [18]. The deglutathionylation of β-actin was shown to have a direct effect on the globular/filamentous actin (G/F) ratio [18]. More recently, the cysteine 253 in NIMA-related kinase 7 (NEK7) has been identified as a target of GSTO1-1 mediated deglutathionylation, a post-translational modification with consequences for NLRP3 inflammasome activation [22].

Less clear is the role of GSTO1-1 in the forward reaction, i.e., the glutathionylation of proteins. This activity has been documented in the presence of high nitrosoglutathione (GSNO) concentrations or glutathione thiyl radicals [18]. In the first case, it was proposed that in the presence of GSNO, GSTO1-1 would be able to promote the formation of the oxidized GSH form (GSSG) as an intermediate in the metabolism of GSNO, and the elevated GSSG levels thus produced could contribute to the increased protein glutathionylation. Moreover, like glutaredoxin, GSTO1-1 is also able to generate GSSG from glutathione thiyl radicals and, through it, to induce protein glutathionylation. These pieces of evidence suggest that GSTO1-1 might contribute to the glutathionylation of proteins in the presence of suitable substrates [18].

Interestingly, together with Cys32, human GSTO1-1 contains three additional conserved cysteine residues (Cys90, Cys192, and Cys237). In a recent study on mouse GSTO1, cysteines were demonstrated to play a critical role in GSTO1 regulation as a sensor of the cellular redox state. In particular, C32A and C236A mutations in the GSTO1 sequence of mice result in significantly different redox-dependent stabilities and enzyme activities [23].

Regarding more classic GST-like activities, GSTO1-1 was reported to have little activity with commonly used GST substrates, such as 1-chloro-2,4-dinitrobenzene (CDNB), 7-chloro-4-nitrobenzo-2-oxa-1,3-diazole (NBD-Cl), trans-octenal, and trans-nonenal [5].

GSTO2-2 has a similar substrate specificity as GSTO1-1 but with significant differences in the substrate/activity profile. The thioltransferase activity of GSTO2-2 (Figure 1a) is similar in magnitude [8], but GSTO2-2 does not exhibit the deglutathionylation activity catalyzed by GSTO1-1 [18] nor S-(phenacyl) the glutathione reductase activity [17]. Regarding the DHA reduction, the reductase activity of GSTO2-2 is approximately 100-fold greater than that of GSTO1-1 [12]. GSTO2-2 is also able to catalyze the reduction of MMA^V^ and DMA^V^, but the latter activity is markedly lower as compared with GSTO1-1 [8,12]. Some differences were reported for more classic GST-like activities, with a high catalytic activity against 1-chloro-2,4-dinitro benzene reported in one study [8] or no detectable activities with common GSTs’ substrates, such as 1-chloro-2,4-dinitro benzene, ethacrynic acid, and cumene hydroperoxide, as shown by others [12].

Finally, beyond their canonical catalyzed reactions, certain GSTs have been suggested to engage in protein–protein interactions, independently of their catalytic functions. This is the case of GSTP1, which can directly interact with intracellular kinases favoring their sequestration and interfering with their interactions [1], and it was suggested that similar functions could also be played by GSTO1-1 [24]. A regulatory mechanism independent of enzyme activities was also proposed for the modulatory function of GSTO1-1 on ryanodine receptors, i.e., channels located in the endoplasmic reticulum membrane responsible for the calcium release [25].

## 3. GSTO Polymorphisms

Several polymorphisms—mostly single nucleotide polymorphisms (SNPs)—have been identified in both coding and non-coding regions of GSTOs’ genes [7,26]. Indeed, DNA sequencing from different ethnic groups allowed the identifications of 31 and 66 polymorphisms in human *GSTO1* and *GSTO2* genes, respectively, with most variations occurring in non-coding regions [26]. To date, however, the effects of coding SNPs have been studied in more detail, and four non-synonymous SNPs have been characterized in each gene, i.e., G95A (rs45529437; C32Y), C419A (rs4925; A140D), G622A (rs11509438; E208K), and C707T (rs11509439; A236V) for *GSTO1* and G121A (rs34400162; V41I), G389A (rs45582439; C130Y), A424G (rs156697; N142D), and C472A (rs34145840; L158I) for *GSTO2*. Moreover, a deletion of codon 155 in the *GSTO1* gene (rs11509437; E155del) was also identified, resulting from a rearrangement of the exon 4/intron 4 splice site and producing a 3 bp deletion.

### 3.1. Allele Frequencies

Studies on different populations demonstrated that there are marked variations in GSTOs’ polymorphisms frequencies and types among ethnic groups [26], but GSTO1*A140D (rs4925), GSTO1*E155del (rs11509437), GSTO1*E208K (rs11509438), and GSTO2*N142D (rs156697) are the most represented [7,27]. Genotype distributions for all polymorphisms were repeatedly reported to be in a Hardy–Weinberg equilibrium [28,29,30,31,32,33,34].

The A140D substitution is the most common GSTO1-1 missense polymorphism with an allele frequency ranging between 0.302 and 0.399 in European populations (German, Spaniard, Italian) [27,35,36] and in Australians with European ancestry [7]; lower frequencies, ranging from about 0.120 to 0.170, were detected in Asian populations (Chinese, Thai, Mongolian, Japanese, Indian) [7,29,37,38,39,40]; and finally, even lower frequencies—ranging from 0.040 to 0.081—were observed in African populations (Bantu, Ovambo) [7,37]. GSTO1*E155del and GSTO1*E208K mutation frequencies never exceeded 10%, with no significant differences among worldwide populations [7,12,27,41]. A linkage disequilibrium was also observed between the GSTO1*E155del and E208K variant in some groups [12,27,32,36]. The little represented GSTO1*A236V substitution, instead, has been described in subjects from Chile and Mexico [26,36].

The N142D substitution is the most frequent *GSTO2* variant in all populations studied [12,26], with a gene frequency of 0.341 among Europeans [27] and 0.310 among Australians with European ancestry [7]; slightly lower frequencies, ranging from 0.173 to 0.285, were detected in Asian populations (Chinese, Thai, Mongolian, Japanese) [7,37,38,40]; and finally, higher frequencies—ranging from 0.583 to 0.855—were observed in African populations (Bantu, Ovambo) [7,37].

### 3.2. Effects of GSTOs’ Polymorphisms: Expression and Functions

Although some studies have focused on the effects of GSTOs’ polymorphisms on gene expression or enzymatic function, the majority have investigated the properties of the most represented variants.

In a study by Mukherjee et al. [26], expression constructs created for all variant allozymes were used to transfect COS-1 cells and evaluate the effects on protein expression. GSTO1-1 polymorphisms A140D (rs4925), E208K (rs11509438), and E155del (rs11509437) did not produce significant effects on protein levels; on the contrary, the A236V (rs11509439) variant was associated with increased levels, whereas the C32Y (G95A) variant produced a reduction greater than 50% of protein levels, caused by an accelerated degradation [26]. Nevertheless, another study on the variant proteins expressed in a strain of *Escherichia coli* showed that the E155del (rs11509437) variant is able to decrease the heat stability of the enzyme [12]. The heightened tendency to unfold, along with a defect in protein refolding, led the authors to conclude that, in some conditions in vivo, the E155del variant would result in the increased turnover and deficiency of GSTO1-1 [42]. Indeed, the same authors found a GSTO1-1 protein deficiency in a T-47D breast cancer cell line *hemizygous* for the E155del variant and a reduction in the GSTO1-1 activity in lymphoblastoid cell lines *heterozygous* for the delE155/K208 haplotype [43]. A similar discrepancy between the two approaches was found for the rarer A236V polymorphism occurring in South American populations: once again, the recombinant protein expressed in a strain of *Escherichia coli* showed a marked instability [36], which contrasts with the findings reported before [26]. From a functional point of view, some studies showed contrasting results about the effects of the GSTO1*A140D polymorphism, causing a nonconservative amino acid substitution from hydrophobic (alanine) to hydrophilic (aspartate) in amino acid 140. In a study by Tanaka-Kagawa [44], the GSTO1*A140D variant was associated with a decrease (about 25%) of thioltransferase activity, as measured with the non-peptidic substrate 2-hydroxyethyl disulphide (HEDS; Figure 1a). On the other hand, no differences between wild-type and GSTO1*A140D variants were found by others [7,12,16,18,21]. Regarding the deglutathionylase activity of GSTO1-1, both GSTO1*A140D and GSTO1*E208K variants showed a significant decrease (Figure 1b) [18], whereas the A140D variant exhibited a higher activity in the forward glutathionylation reaction with glutathione thiyl radicals as a substrate [18]. Finally, the A140D polymorphism did not modify the 1-chloro-2,4-dinitrobenzene (CDNB) metabolism or the S-(phenacyl)glutathione, DHA, MMA^V^, or DMA^V^ reductase activity [12,16,17,44].

Regarding other GSTO1-1 polymorphisms, E155del increases the GSH conjugation with CDNB, thioltransferase, MMA^V^, and DMA^V^ reductase activities, possibly due to the increased flexibility of the mutant protein towards different substrates [7,12,16], whereas A236V is associated with diminished (80–90%) thioltransferase, DHA, MMA^V^, DMA^V^, and S-(phenacyl)glutathione reductase activities [6,36]. The E208K variants slightly increased thioltransferase, MMA^V^, and DMA^V^ reductase activities, with no effects on the DHA reductase activity [12]; on the contrary, it significantly reduced the deglutathionylase activity [18]. Finally, in a study by Tanaka-Kagawa [44], the still poorly studied GSTO1*T217N (C650A) variant was found to significantly reduce both the thioltransferase and MMA^V^ reductase activity. However—as suggested by Whitbread et al.—the T217N variant has not been detected in any population survey and must be very rare or the result of a sequencing error [16,29].

The effects of GSTO2-2 polymorphisms have been less thoroughly studied. The expression constructs used to transfect COS-1 cells revealed that C130Y (rs45582439) and L158I (rs34145840) variants significantly reduced protein production (50% and 80%, respectively), whereas N142D (rs156697) and V41I (rs34400162) produced a reduction of about 20%. Once again, an accelerated protein degradation was proposed as the possible underlying mechanism [26]. However, lower levels of GSTO2-2 transcript were detected in both the cerebellum and temporal cortex of autopsied subjects with and without Alzheimer disease and the N142D (rs156697) polymorphism [45]. Therefore, it is not surprising that polymorphisms in GST omega-class members have been investigated in relation to several clinical disorders, including Parkinson’s disease, vascular dementia, stroke, amyotrophic lateral sclerosis, chronic obstructive pulmonary disease [21], and cancer [46].

From a functional point of view, the N142D substitution in GSTO2-2, inducing a nonconservative amino acid change from an uncharged polar (Asn) to an acidic amino acid (Asp), does not influence the specific activity of the enzyme with any substrate. [7,12]. On the contrary, the functional relevance of the other GSTO2*A183G (rs2297235) polymorphism has not been fully ascertained, although it has been investigated in some studies focused on urinary arsenic concentrations and arsenic-related neoplasms [47,48,49].

The effects of the main polymorphisms on GSTO1-1 and GSTO2-2 functions are summarized in Table 2.

## 4. GSTOs’ Polymorphisms and Cancer

**Figure 2:**
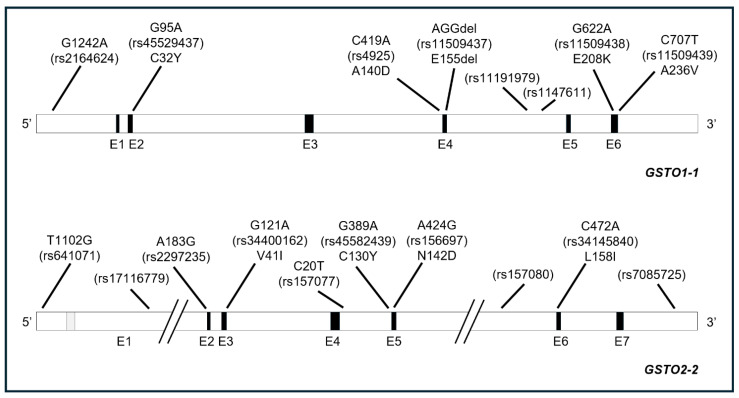
The schematic localization of major SNPs in GSTOs’ sequences investigated in cancer studies. Both GSTOs’ sequences contain six exons encoding the open read frame (GSTO1: E1-E6; GSTO2: E2-E7; black rectangles).

Many studies have explored the association between SNPs in the omega-class GSTs and the susceptibility to various cancers (Figure 2) [21,50]. Clinical association studies have shown that genetic alterations within the human GST isozymes may play a key role in cancer susceptibility and treatment [51]. GSTO1-1 upregulation has been reported in various cancers, including pancreatic cancer [52], esophageal adenocarcinoma [53,54], colorectal cancer [55], and ovarian cancer, where the upregulation has been associated with drug resistance [24,56]. GSTO1-1 was also demonstrated to modulate key intracellular signaling pathways involved in cell survival and proliferation, such as ERK1/2, JNK, and AKT [24,57,58,59]. Additionally, it plays a role in regulating antioxidant defense mechanisms through its dehydroascorbic acid reductase activity, which is critical for maintaining the redox balance within the cells [11]. This dual function underscores the enzyme’s importance in both promoting cellular survival and defending against oxidative stress, processes that are closely tied to cancer development and progression.

**Figure 3:**
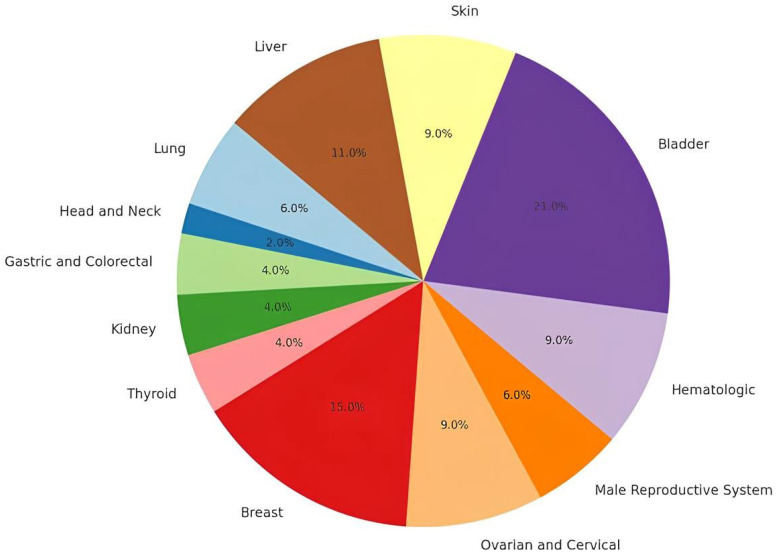
Percentage of studies investigating GSTOs’ polymorphisms expressed by type of cancer.

As expected, GSTO1*A140D, GSTO1*E155del, and GSTO2*N142D are the most common polymorphisms that have been investigated in cancer, but results remain inconsistent, as underlined in a previous meta-analysis performed by Xu et al. [46]. This meta-analysis of 20 studies suggests that GSTO2*N142D may significantly increase the cancer risk in the Caucasian population, particularly for breast cancer, while GSTO1-1 polymorphisms show no significant association to cancer susceptibility. However, in recent years, additional studies have emerged addressing this specific topic. The aim of the final section of this systematic review is to summarize and examine both older and more recent findings on GSTOs’ polymorphisms in human cancer, categorized by cancer type (Figure 3; Table 3).

Articles published between the early 2000s—the years of the first references of GSTOs’ polymorphisms in cancer—and June 2025 were identified through a computerized search on the MEDLINE (National Library of Medicine Bethesda MD) and PubMed database. The search strategy employed a combination of keywords, including [(cancer) AND (GST)], [(cancer) AND (glutathione transferases)], [(cancer) AND (GSTO)], [(cancer) AND (glutathione transferases omega)], and [(cancer) AND (GSTO) AND (polymorphism)]. Additional combinations included specific cancer types with GSTO-related polymorphisms to ensure a comprehensive literature coverage. Studies were included if they investigated GSTOs’ polymorphisms either as the main topic of the study or as part of a broader gene panel and if they explored associations with the severity, mortality, or disease progression of a type of cancer. Publications that were not in English, incomplete texts, and these were excluded.

A first point to highlight is that some types of neoplasms have been the object of numerous studies. Indeed, GSTs are primarily perceived as enzymes involved in detoxication processes, and therefore it is not surprising that a large part of epidemiological studies have focused on cancers related to environmental factors, including cigarette smoking or occupational toxicants. Chemical carcinogens may require metabolic activation by phase I enzymes, such as cytochrome P450 oxidase, and/or phase II enzymes, such as GSTs, and it is conceivable that SNPs in genes encoding these enzymes might contribute to the inter-individual susceptibility to various malignancies.

As stated above, GSTO1-1 and GSTO2-2 have MMA^V^ and DMA^V^ reductase activity and are involved in the biomethylation of inorganic arsenic [12,14,15]. A chronic exposure to arsenic in drinking water, even at very low concentrations, has been strongly associated with a variety of neoplasms [60], including skin, bladder, lung, liver, and testicular germ cell cancers [61,62] and cardiovascular diseases [63]. High levels of arsenic are mainly found in groundwater obtained from Bangladesh, India, China, parts of the United States, small areas of Argentina, Australia, Chile, Mexico, Vietnam, and Taiwan [49,61,64]. The biotransformation of arsenic involves alternating methylation and reduction reactions, where the reduction of MMA^V^ is the rate-limiting step. The inter-individual and genetic variability in arsenic metabolism and clearance is thus a topic of interest, particularly in relation to the cancer incidence in arseniasis areas [47,65,66].

As regards GSTOs, several studies have investigated the potential associations between chronic arsenic exposure, *GSTO1*, and *GSTO2* genotypes, and urinary arsenic profiles (see [21] for refs). In brief, a first study by Marnell [67] revealed an association between increased urinary inorganic arsenic and heterozygous GSTO1*E155del and GSTO1*E208K polymorphisms in Mexican subjects chronically exposed to arsenic in their drinking water (9–100 microg/L). However, this result was not confirmed in a following study on Chilean subjects [36]. On the other hand, in a study on residents in Bangladesh, homozygous wild types for GSTO1*A140D (rs4925) and GSTO2*A183G (rs2297235) were significantly associated with higher MMA and DMA urinary concentrations [47]. Notably, a large part of other studies (see [21] for refs) focused only on GSTO1*A140D and GSTO2*N142D polymorphisms, i.e., variants not supposed to change MMA^V^ or DMA^V^ reductase activities [12]. Indeed, although some relatively weak associations have been identified, most of the studies did not find any significant association between GSTO1/GSTO2 polymorphisms and urinary inorganic arsenic levels. It must be considered that each step in the biotransformation of inorganic arsenic may have alternative enzymes [68], making it plausible that large inter-individual differences in urinary inorganic arsenic levels among chronically exposed subjects are not solely attributable to GSTOs’ polymorphisms [21].

### 4.1. Bladder Cancers

Urothelial carcinoma (UC) includes cancers of the bladder, renal pelvis, and ureter. Major groups of studies about GSTOs’ polymorphisms have been focused on bladder cancers—as arsenic is bioconcentrated in urine—and were mainly from countries with high levels of arsenic contamination of the groundwater [48,65,69,70,71,72,73,74,75,76]. Indeed, cigarette smoking, occupational exposure to certain chemicals, and chronic infections together with exposure to arsenic through drinking water are known risk factors for bladder cancer [73].

A major number of studies were from Taiwan. In a first report by Wang and collaborators [69], a significant increase in the risk of UC was observed for subjects carrying the G/G genotype of GSTO2*A183G, a substitution identified at the 5′ untranslated (5′UTR) gene region of GSTO2 (rs2297235), whereas a higher, but not statistically significant, risk was detectable for the more common GSTO2*N142D (A424G) and GSTO1*A140D (C419A) polymorphisms. On the other hand, the analysis of the haplotypes/diplotypes of GSTO1 and GSTO2 revealed that the diplotype combining GSTO1*C419/GSTO2*A183/GSTO2*G424 had a significantly higher risk of urothelial carcinoma, as compared with other diplotypes. The risk is further increased when the CYP2E1 genotypes and environmental risk factors, such as cigarette smoking, alcohol consumption, arsenic, and occupational exposures, are also considered.

Lesseur et al. [71] found that the bladder cancer risk overall was associated with the GSTO2*N142D polymorphism. On the contrary, another study by Chung [70] focused on GSTO1*A140D (C419A), GSTO1*E208K (G622A), and GSTO2*N142D (A424G) polymorphisms showed a protective effect of the homozygous GSTO2*N142D polymorphism on the UC risk. No association was observed for GSTO1 polymorphisms.

The following study by Hsu was on a large cohort and found a significative association between the homozygous GSTO1*A140D variant and the UC risk, although it was limited to subjects exposed to very high arsenic levels (cumulative arsenic exposure ≥20 mg/L*year). Moreover, subjects with the GSTO AGG/AGG diplotype of GSTO1*A140D (C419A), GSTO2 5′UTR (-183) A/G, and GSTO2*N142D (A424G) had a 34-fold higher cancer risk when exposed to high levels of arsenic [48].

Finally, a final study by Tung [73] focused on the possible association between cigarette smoking, GSTOs’ polymorphisms, and UC cancer. Cigarette smoking is considered the major exogenous risk factor for the development of bladder cancer [77]. An increased risk of UC was found in never-smoker subjects expressing the homozygous wild-type GSTO1*A140; moreover, an even more increased UC risk was found in heavy smokers carrying the same genotype.

The GSTOs’ polymorphisms and bladder cancer association was also investigated in three studies from the USA. In the paper by Beebe-Dimmer [65], subjects were selected from southeastern Michigan, where approximately 8% of the population was exposed to arsenic concentrations exceeding the Maximum Contaminant Level (MCL) of 10 μg/L. Contrary to the study by Hsu on Taiwanese subjects [48], authors found a positive association between arsenic exposure (the uppermost quartile, 3.72 μg/L in drinking water) and bladder cancer in subjects homozygous for the wild-type GSTO1*A140 variant. A second study by Lesseur [71] focused on subjects from New Hampshire who were exposed to arsenic levels in water beyond 10 μg/L. Among GSTOs, only the GSTO2*N142D (A424G) was analyzed, and a significative association of the minor variant with the bladder cancer risk was found, independently of an arsenic exposure. Finally, Ke et al. [76] investigated 114 SNPs of 21 genes involved in the glutathione pathway and their possible association with non-muscle invasive bladder cancer recurrence in patients treated with a transurethral resection alone (TUR) or in combination with intravesical bacillus Calmette Guérin instillation (BCG) therapy. However, no significant association was found with cancer recurrence and GSTOs’ polymorphisms, including GSTO1 rs4925, rs11509438, GSTO2 rs641071, rs156697, and rs3740466.

Another couple of studies about bladder cancers and GSTOs’ polymorphisms were from Serbia. In the first study on muscle invasive bladder cancer patients [72], both homozygous mutant GSTO1*C419A (A140D) and GSTO2*A424G (N142D) genotypes were independent predictors of a higher risk of death. Moreover, a shorter survival was detectable in a subgroup of patients receiving chemotherapy and who were homozygous for the GSTO2*A424G (N142D) variant. In a second study by the same group [74], homozygous carriers of the mutant GSTO2*A424G (N142D) genotype were found to be at a higher risk of transitional cell carcinoma (TCC) of the urinary bladder in comparison with carriers of at least one wild-type GSTO2 allele, and the risk was even higher when such a polymorphism was associated with cigarette smoking. Moreover, the concomitant presence of GSTO1 wild-type and mutant GSTO2 genotypes (GSTO1*C/GSTO2*G haplotype) was associated with the highest risk of developing TCC.

A last study was performed in China on a group of subjects diagnosed with non-muscle invasive bladder cancer [75] and focused on the possible influence of GSTOs’ genetic variants on the clinical outcomes and toxic effects of chemotherapy. Patients were instilled with chemotherapeutic drugs (epirubicin or mitomycin) after the transurethral resection of the bladder tumor, and data were collected. Subjects treated with epirubicin, expressing homozygous wild-type *GSTO1* (GSTO1 A140, GSTO1 CC genotype), had a lower risk for bladder cancer recurrence (RFS) and a longer mean survival time (MST), as compared with the GSTO1 AC+AA genotype. Homozygous wild-type *GSTO1* was also associated with a reduced risk of toxicity (i.e., hematuria) in patients instilled with epirubicin. No significant association was found between the *GSTO1* genotypes and mitomycin treatment or between the GSTO2 polymorphism and all the parameters analyzed.

As also summarized in Table 3, the results on bladder cancer are quite contrasting. However, different ethnicities and types of bladder cancer are variables to take into consideration. GSTs are included among the low-penetrance genes contributing to cancer risk; i.e., they can contribute to the development of a type of cancer in association with environmental factors. Currently, only a some of these studies took into consideration arsenic exposure [48,65,69,71], smoking [72,73], or chemotherapy [75], and only three studies focused on GSTOs’ haplotypes/diplotypes rather than on a single polymorphism [48,69,74]. Finally, some studies solely evaluated the risk of cancer while others focused on the cancer-related risk of death.

### 4.2. Skin Cancers

The possible connection between GSTOs and arsenic metabolism is the background for a group of four studies focused on skin cancers. Arsenic-related skin lesions—including, e.g., keratosis on palms and soles, hypopigmentation, and characteristic raindrop-like pigmentation—may indeed appear after short periods of arsenic exposure and give rise to the majority of arsenic-induced basal and squamous cell skin cancers (e.g., [78]). In an initial study on the Bangladeshi population [79], the homozygous GSTO1*A140D polymorphism (GSTO1 AA genotype) was related to an elevated risk of skin lesions, whereas the two other variant alleles evaluated, namely GSTO1*E208K (rs11509438) and GSTO1*E155del (rs11509437), were related to a reduced risk of skin lesions. Furthermore, subjects with the AAG/AAG diplotype (i.e., the diplotype containing the high-risk alleles for all three SNPs) had an elevated risk for arsenic-related skin lesions. The authors also found that the association between GSTO1 genotypes and the risk of skin lesions differed by the percentage of monomethylarsonous acid (%MMA) in urine, suggesting the additivity of genotypes/diplotypes with the water arsenic concentration and urinary %MMA on the association observed. On the other hand, a second study from India focused on the arsenic-exposed population of West Bengal [39] did not find any significant association between GSTO1-1 or GSTO2-2 variants and arsenic-induced skin lesions. The third study is from Brazil [80] and thus focused on a different geographic and ethnic groups of subjects with a highly heterogeneous background. Among GSTOs, only the GSTO2*N142D polymorphism was evaluated in subjects affected by different types of skin cancers, i.e., squamous cell carcinomas, basal cell carcinoma, malignant melanomas, and non-malignant lesions (benign). However, no significant difference was detectable for the rate of the GSTO2-2 variant in the different types of malignant tumors studied.

A last study focused on subjects from areas of northwest China with high levels of arsenic in the drinking water (969 μg/L) [49]. Different GSTO polymorphisms were analyzed, namely rs11191979, rs2164624, rs2282326, and rs4925 for GSTO1-1 and rs156697 and rs2297235 for GSTO2-2. Allele frequencies, genotypes, and haplotypes were evaluated, and significant associations with a high risk of arsenic-related skin lesions were found for both more common and less studied GSTO polymorphisms (see Table 3). As regards the GSTO1*A140D (C419A; rs4925) polymorphism, carriers of at least one variant allele (*A) had an increased risk of skin lesions. As far as *GSTO2*, carriers of the AG genotype for the GSTO2*N142D (A424G; rs156697) and *GSTO2* polymorphism in the 5′UTR gene region (A183G; rs2297235) were at a higher risk as well.

### 4.3. Liver Cancers

Considering the involvement of GSTs in phase II detoxification reactions and their high hepatic expression [5,7], it is quite surprising that only a few studies focused on GSTOs’ polymorphisms in liver cancers. A first study on subjects from Thailand [38] investigated four different types of cancer, namely hepatocellular carcinoma (HCC), cholangiocarcinoma (CCA), colorectal cancer, and breast cancer. About 30 patients for all the types of cancer were recruited, and the rates of GSTO1*A140D (rs4925) and GSTO2*N142D (rs156697) polymorphisms were evaluated. Authors found a significantly different genotypic distribution for the GSTO1*A140D variant in HCC, cholangiocarcinoma, and breast cancer, as compared to the control group. No significant differences were observed for GSTO1*A140D in colorectal cancer and GSTO2*N142D in all cancer types.

Another study on Chinese subjects [81] examined rare GSTOs’ variants and found that the *GSTO2* polymorphism (rs7085725) located in the 3′-UTR of the gene was significantly associated with overall survival (OS) in HCC, with subjects carrying at least one variant allele having an increased risk of death and a shorter median survival time. Moreover, the same carriers were more susceptible to smoking exposure.

Wang et al. [82] investigated three well-defined polymorphisms (rs156699, rs157077, and rs7085725) within the GSTO2-2 gene to determine their potential to predict OS among 228 HCC patients undergoing a transarterial chemoembolization (TACE) treatment. Only one of three polymorphisms examined, rs157077, was significantly associated with the OS of TACE-treated HCC (*p* = 0.003), and its mutant allele conferred a higher risk of death than its wild homozygotes, by suggesting that rs157077 may play a role prognostic predictions for Chinese HCC patients undergoing TACE therapy.

In the study by Shaban et al. [83] on Egyptian subjects, an additional environmental factor not previously explored was considered. The authors focused on hepatitis B virus (HBV) infection-associated liver diseases and found that both homozygous and heterozygous wild-type GSTO1*A140 (i.e., A/C and C/C genotypes) were more prevalent in all the patient groups analyzed, including normal HBV carriers (N) and acute (A), chronic (CH), cirrhosis (CI), and hepatocellular carcinoma (HCC) patients, compared to healthy controls. On the contrary, the same patients showed a significantly lower frequency of the homozygous GSTO1*A140D variant (i.e., A/A genotype). Similarly, for GSTO2-2 both homozygous and heterozygous GSTO2*N142D variants (i.e., A/G and G/G genotypes) were more represented in all patient groups compared to controls, while these patients exhibited a significantly lower frequency of the homozygous wild-type GSTO2*N142 (i.e., A/A genotype). Overall, subjects carrying both the wild-type GSTO1*A140 and the GSTO2*N142D variant allele were more susceptible to HBV disease progression.

Finally, in a study by Chaiteerakij [84], the association between SNPs of inflammation or cancer-associated genes—including the GSTO1*A140D variant—and the CCA risk and survival was investigated in a cohort of patients and healthy controls from Minnesota (USA). The authors found no significant associations between the allele or genotype frequencies of any of the tested SNPs and survival outcomes, even when cancers were classified by subtype (intrahepatic, extrahepatic CCA).

### 4.4. Lung Cancers

Cigarette smoking remains the dominant factor in both chronic obstructive pulmonary disease (COPD) and lung cancer, but GSTOs’ polymorphisms may modify an individual’s susceptibility by influencing how well their body handles oxidative stress and the detoxification of cigarette smoke components. A genome-wide association analysis as part of the Framingham heart study reported that the GSTO2 N142D polymorphism was associated with both lower levels of the lung function parameters, i.e., the forced expiratory volume in one second (FEV1) and forced vital capacity (FVC) [28]. This association was confirmed in a large study of over 8000 subjects from the northern population of the Netherlands (LifeLines cohort study), where there were significant interactions between the exposure to environmental tobacco smoke, GSTOs’ polymorphisms, and FEV1 [85]. Another study of 355 COPD cases and 195 apparently healthy (ex)smoking controls failed to find an association between the GSTOs’ polymorphisms and FEV1 but found that the GSTO2 N142D polymorphism and the *GSTO1 140D/GSTO2 142D* haplotype are associated with an increased risk of COPD [86].

However, a couple of studies from the USA focused on lung cancers. The first study involved subjects with COPD and COPD with lung cancer [87]. Authors analyzed 470 SNPs from 52 genes involved in glutathione metabolism, DNA repair, and inflammatory response pathways selected based on HapMap data of 60 unrelated Caucasian (CEU) subjects (Release 22/Phase II on NCBI B36) using Haploview. Among them, five GSTO1-1 polymorphisms (rs11191975, rs1147611, rs11509438, rs2164624, rs628480) and six GSTO2-2 polymorphisms (rs10491045, rs11191990, rs156699, rs157077, rs157080, rs568526) were identified. The authors then evaluated the cumulative effects of the statistically significant SNPs on the COPD risk. Five of the fifty-two genes were found to be significantly associated with COPD. A strong association of the GSTO2-2 gene and COPD with lung cancer was detected, suggesting that GSTO2-2 may be critical for developing cancer among patients with COPD.

On the other hand, a large study on patients with pathologically confirmed primary lung cancer, including both small cell and non-small cell lung cancer, and investigating 290 SNPs in the glutathione pathway did not find any significant association between different GSTO1-1 (rs2164624; rs4925) or GSTO2-2 (rs156697) polymorphisms and survival upon cisplatin chemotherapy [88]. Similarly, a Turkish study on non-small cell lung cancer (NSCLC) did not find any significant association between the GSTO1*A140D variant and the risk of two histological subtypes of NSCLC, even in smokers [89]. Unlike tumors of different localizations, these three studies seem to agree in finding no correlations between lung tumors and the GSTOs’ polymorphisms examined.

### 4.5. Head and Neck Cancers

Head and neck cancers include cancers of the oral cavity, pharynx, and larynx. They arise due to a combination of environmental factors and inherited factors, including genetic polymorphisms that affect the ability to metabolize environmental carcinogens [90]. Considering the role of GSTs in detoxification, a study on Thai subjects focused on GSTO1*A140D and GSTO2*N142D polymorphisms in head and neck squamous cell carcinoma (HNSCC) risk [91]. The authors found no significant association between the two GSTO polymorphisms and the risk of HNSCC. On the other hand, an association between the GSTO1*A140 wild genotype with a susceptibility for nodal metastasis and an advanced tumor stage was found, leading to the suggestion that the GSTO1*A140D variant may have a protective role against HNSCC aggressiveness.

### 4.6. Gastrointestinal Cancers

A couple of studies from the same group focused on gastrointestinal cancers in Iranian patients. In a first study on gastric cancers [92], authors found no association between the GSTO2*N142D polymorphism and cancer risk. On the other hand—when compared to the other two genotypes (i.e., N/N, N/D)—the homozygous GSTO2*N142D (D/D) genotype was found to be associated with a lower risk of gastric cancer in subjects without a history of cancer in their first-degree relatives. In a further study on colorectal cancer [93], no association was found between the same GSTO2*N142D polymorphism and cancer risk. Here, subjects with the homozygous wild-type GSTO2*N142 genotype (N/N) and with a positive family history were found to be at a high risk for developing colorectal cancer, when compared to subjects with other genotypes (i.e., D/D or N/D) and a negative family history of cancer.

A lack of an association between the GSTO2*N142D polymorphism and colorectal cancer risk was also found in the study by Marahatta on Thai subjects [38], together with no significant association between the GSTO1*A140D polymorphism and the risk of the same type of cancer.

### 4.7. Kidney Cancers

Radic et al. [94] investigated the impact of specific genetic variants—GSTO1*A140D (C419A; rs4925), GSTO2*N142D (A424G; rs156697), and the *GSTO2* polymorphism in the 5′UTR gene region (A183G; rs2297235)—on the risk of developing the most aggressive renal cell carcinoma (RCC) subtype, the clear cell RCC (ccRCC). The study analyzed these variants both independently and in interaction with established risk factors (smoking, obesity, and hypertension) in a cohort of 239 Serbian patients. The analysis revealed that subjects with combined variant GSTO1*A/A (GSTO1*A140D; D/D) and GSTO2*G/G (GSTO2*N142D; D/D) genotypes had a higher risk of developing cancer. Furthermore, a haplotype analysis confirmed that the presence of one copy of the variants GSTO1*A (rs4925), GSTO2*G (rs156697), and GSTO2*G (rs2297235) was associated with an increased risk of ccRCC. Similarly, the presence of the only homozygous variant GSTO2*G/G (rs156697) in the subgroup of smokers was associated with a higher risk. Again, the combinations of allelic variants—rather than the single variant—of GSTOs and the inclusion of environmental factors seems to better define the potential prognostic value of GSTOs.

A further follow-up study by Radic et al. [95] explored the same three polymorphisms and their effects on the postoperative prognosis in patients with ccRCC. They found that the GSTO1*C/C wild-type genotype (GSTO1*A140; A/A) was significantly associated with a shorter survival but only in male carriers. Moreover, this genotype independently predicted a higher overall mortality risk among male ccRCC patients. In contrast, no significant effects of either *GSTO1* (rs4925) or *GSTO2* (rs156697 and rs2297235) polymorphisms were observed on the OS among female ccRCC patients.

### 4.8. Thyroid Cancer

In a study on Brazilian patients [96], the authors did not find any association between the GSTO1*A140D polymorphism and clinico-pathological features of thyroid nodules in subjects with follicular carcinomas, papillary carcinomas, follicular adenoma, and multinodular goiters. This result was further confirmed in a following study by the same group [97] on a larger cohort of patients with benign goiters, follicular adenomas, and papillary and follicular carcinomas. The GSTO1*A140D polymorphism alone or in combinations with polymorphisms of other genes did not correlate with any of the clinico-pathological features selected.

### 4.9. Breast Cancer

Numerous studies have investigated GSTO polymorphisms in relation to breast cancer [30,31,38,98,99,100,101,102]. These studies highlight that polymorphisms in low penetrance genes—including GSTs—in association with environmental factors significantly contribute to the tumorigenesis [103].

In the already mentioned study by Marahatta on a small group of subjects from Thailand [38], authors found a significantly higher gene frequency of the GSTO1*A140D variant (heterozygous or homozygous carriers) in breast cancer patients, as compared to the control group. No association was found for the GSTO2*N142D polymorphism. However, in another study from Thailand including premenopausal and postmenopausal women [99], no association between *GSTO1* and *GSTO2* genotypes and the risk of breast cancer was found, although a higher prevalence of the homozygous wild-type GSTO1*A140 allele (A/A) was found in advanced-stage breast cancer.

A couple of studies on Iranian patients, instead, produced partially different results. In the first study by Masoudi, the lack of an association between the breast cancer risk and GSTO2*N142D (A424G; rs156697) polymorphism was again confirmed [101]. Nevertheless, in the study by Sharif [102], the authors found significant associations between the breast cancer risk and allele frequencies of both the variant A allele of GSTO1*A140D (C419A; rs4925) and the G allele of GSTO2*N142D (A424G; rs156697). A meta-analysis of 20 studies involving 4770 cases and 5701 controls revealed a significant increase in cancer susceptibility in Caucasian populations linked to the *GSTO2* polymorphism (A424G; rs156697), but no significant association was found for the *GSTO1* polymorphism. Additionally, a subgroup analysis showed that the GSTO2 polymorphism was significantly associated with an elevated risk of breast cancer (GG vs. AA), specifically in the homozygote, recessive, and allelic comparison model [46].

Other studies have then attempted to identify suitable subgroups among recruited patients. In a study from Denmark, the authors limited the recruitment of patients to postmenopausal women [98] and found an association between the homozygous GSTO1*A140D variant and breast cancer risk but only in estrogen receptor-positive cases. No association was detectable for estrogen receptor negative cancers. On the other hand, another large population study on postmenopausal breast cancer patients from Germany [30] investigated 109 common SNPs in 22 oxidative stress-related genes and, among them, GSTO1*A140D polymorphism was not associated with overall mortality neither with possible differential effects by radiotherapy treatment.

Sohail et al. [31] investigated the association of the GSTO2-2 (rs156697; N142D) polymorphism with the risk of breast cancer in the Pakistani population. The results indicated that the presence of heterozygous GSTO2-2 (Asn/Asp) was significantly higher in breast cancer cases than in the control group. However, neither the heterozygous nor the wild-type GSTO2-2 genotype showed a significant overall association with breast cancer risk. Additionally, postmenopausal women with either heterozygous or wild-type GSTO2-2 genotypes had a lower breast cancer risk compared to those with the homozygous mutant *GSTO2* genotype. However, the presence of both GSTP1 and GSTO2 polymorphisms together was also found to be associated with an increased risk of breast cancer, particularly in premenopausal women. Therefore, in postmenopausal patients, an increased breast cancer risk is linked to the mutant GSTO2 genotype alone, whereas in premenopausal women, the combined presence of GSTP1 and GSTO2 may further elevate the risk.

Finally, the large study by Andonova et al. on German patients [100] did not find any significant association between breast cancer risk or histopathological tumor characteristics and the two major GSTO polymorphisms—namely, GSTO1*A140D (C419A; rs4925) and GSTO2*N142D (A424G; rs156697)—or the two minor polymorphisms localized in the 5′UTR gene region of GSTO1-1 (G1242A; rs2164624) and GSTO2 (A183G; rs2297235), respectively. The lack of an association was verified both in the general population and in a different subgroup considering their menopausal status, family history of breast cancer, use of oral contraceptives, use of hormone therapy, body mass index, and smoking.

### 4.10. Ovarian and Cervical Cancers

Three studies addressed GSTO polymorphisms in ovarian cancer [104,105,106]. In the study by Morari on Brazilian patients [104], no differences were found between the GSTO2*N142D variant and the risk of ovarian cancer, whereas the same polymorphism was associated with an increased risk of ovarian cancer in a study on a small group of Thai patients [105]. More recently, a study of Serbian subjects [106] confirmed that homozygous carriers of the GSTO2*N142D variant have an increased risk of ovarian cancer, whereas carriers of the H4 haplotype (i.e., GSTO1*A variant allele and GSTO2*A wild-type allele) have a lower OC risk.

Only one study focused on GSTO1-1 and GSTO2-2 in cervical cancer in Iranian women [107]. The authors found that carriers of the GSTO1*D140 variant (GSTO1*A allele; rs4925), heterozygous GSTO1*A140D (*C/*A genotype), and the combination of heterozygous GSTO1*A140D (140AD) and homozygous wild-type GSTO2*N142 (142NN) had a lower susceptibility to human papillomavirus (HPV) 6, 16, 18, and 16/18 infections and to cervical cancer.

### 4.11. Male Reproductive System Cancers

Male reproductive system cancers, also known as male urological cancer, include any malignancies that originate in the men’s reproductive or urinary tract organs. The two most common types are prostate cancer and testicular cancer.

Environmental and lifestyle factors, including pollutants, smoking, and diet, are considered potential contributors to the risk of prostate cancers, alongside geographical and individual factors [108,109]. Once again, in this type of cancer, polymorphisms of enzymes involved in phase I and phase II reactions, including GSTs, were thus deeply investigated, and, indeed, GSTO1-1 was physiologically found to be highly expressed in the prostate [5]. In a study by Lima on Brazilian patients [110], no differences were found between the GSTO1*(A140D) allele frequency or genotypes in prostate cancer patients when compared to subjects with benign prostatic hyperplasia. Additionally, no association was found with the parameters of aggressiveness or the response to radiotherapy.

On the contrary, in a study by Santric on Serbian subjects [111], carriers of homozygous GSTO1*A/A (rs4925; D/D) and GSTO2*G/G (rs156697; D/D) variant genotypes had a higher risk of developing prostatic cancer, but no association with overall survival was found.

Germ cell tumors (GCTs) are malignancies most frequently arising in the gonads, and, interestingly, GSTO2-2 is physiologically highly expressed in the testis [16]. In the only pilot study on Serbian patients, the association between GSTO1*A140D (C419A; rs4925), GSTO2*N142D (A424G; rs156697), and *GSTO2* polymorphisms in the 5′UTR gene region (A183G; rs2297235) and the risk for GCT development was investigated [34]. In this study, the authors found that carriers of the GSTO1*C/A*C/C (rs4925; A/D, D/D) and of the GSTO2*A/G*G/G (rs2297235) genotypes exhibited a heightened risk of developing testicular GCTs. Moreover, subjects expressing combined GSTO2*A/G*G/G (rs156697; N/D, D/D) and GSTO2*A/G*G/G (rs2297235) genotypes had a higher risk for testicular GCTs. Therefore, this pilot study suggests that GSTO polymorphisms may influence the antioxidant activity of GSTO isoenzymes, potentially increasing the susceptibility to testicular GCTs in affected individuals.

### 4.12. Hematologic Cancers

Acute lymphoblastic leukemia (ALL) is the most common type of malignancy in children. In a study on Thai subjects [40], the authors found a significant association between the heterozygous GSTO1*A140D (rs4925) variant and ALL risk. Moreover, the heterozygous GSTO2*N142D (rs156697) polymorphism was significantly associated with the high-risk group of ALL, according to the ALL patient’s classification.

On the contrary, in a case–control study Rezazadeh et al. [112] investigated the association between GSTO1*A140D or GSTO2*N142D polymorphisms and the susceptibility to Pre-B ALL in Iranian child populations, by showing a similar genotype distribution among the Pre-B ALL patients and the healthy controls. Therefore, there was no significant association between these variants and reducing the risk of childhood Pre-B ALL. However, only the combined effects of the 140AA/142DD and 140DD/142NN polymorphisms on reducing the Pre-B-ALL risk were reversely correlated.

Another study from India on B-ALL [113] documented no association between the allele or genotype frequency of the GSTO1*A140D variant and cancer risk. As regards GSTO2*N142D, a significative association of the B-ALL risk was found for the frequency of the variant G allele (D140) and for heterozygous GSTO2-AG (N/D) and homozygous GSTO2-GG (D/D) variant genotypes. Among the haplotypes, GSTO1*C/GSTO2*G was significantly associated with B-ALL risk. Moreover, the GSTO2*GG (D/D) genotype was found to be a poor and independent prognostic factor for disease-free survival.

Finally, a report on the aggressive mantle cell lymphoma (MCL) in patients treated with R-HyperCVAD over a ten-year follow-up identified significant associations between the overall survival and three *GSTO1* polymorphisms (rs1147611, rs4925, rs2164624) as well as two *GSTO2* polymorphisms (rs156697, rs157080) [114].

**Table 3:** A general list of the GSTO polymorphisms and cancer risk in different types of malignant tumors.

Type of Cancer	Country	Number of Patients and Control Subjects	Main Findings	Ref.
Urothelial bladder carcinoma (UC)	Taiwan	764 DNA specimens from a long-term follow-up cohort in southwestern Taiwan	Polymorphisms: GSTO1*A140D (C419A; rs4925), GSTO1*E155del (AGG deletion; rs11509437), GSTO1*E208K (G622A; rs11509438), GSTO1*T217N (C650A; rs15032), GSTO2 5′UTR (-183) (A183G; rs2297235), GSTO2*N142D (A424G; rs 156697) Main findings: Increased UC risk in subjects exposed to high arsenic concentrations (cumulative arsenic exposure (CAE) ≥ 20 mg/L*year) and carrying - GSTO1*A140D homozygous variant; - diplotype AGG/AGG of GSTO1*A140D (C419A), GSTO2 5′UTR (-183) (A183G; rs2297235) and GSTO2*N142D (A424G; rs 156697).	[48]
Bladder cancer	USA	219 patients vs. 273 controls	Polymorphisms: GSTO1*A140D (C419A; rs4925), intron variant GSTO-1 (A/C; rs2282326) Main findings: - no significant associations between GSTO-1 polymorphisms and bladder cancer risk overall; - positive association between arsenic exposure and bladder cancer among subjects carrying the homozygous wild-type GSTO1*A140D.	[65]
Urothelial bladder carcinoma (UC)	Taiwan	520 UC cases vs. 520 controls	Polymorphisms: GSTO1*A140D (C419A; rs4925), GSTO2*N142D (A424G; rs156697), GSTO2 polymorphism in 5′UTR gene region (A183G; rs2297235) Main findings: - increased risk of UC for subjects carrying the G/G genotype of GSTO2*A183G; - increased risk of UC for the diplotype GSTO1*C419/GSTO2*A183/GSTO2*G424; - joint effects of GSTO1, GSTO2, and CYP2E1 genotypes and environmental factors (cigarette smoking, alcohol consumption, arsenic, and occupational exposures) on UC risk.	[69]
Urothelial bladder carcinoma (UC)	Taiwan	149 UC cases vs. 251 controls	Polymorphisms: GSTO1*A140D (C419A; rs4925), GSTO1*E208K (G622A; rs11509438), GSTO2*N142D (A424G; rs156697) Main findings: - reduced risk of UC for subjects carrying the homozygous GSTO2*N142D variant; - no association between GSTO1*A140D or GSTO1*E208K and UC risk.	[70]
Bladder cancer	USA	832 patients vs. 1191 controls	Polymorphisms: GSTO2*N142D (A424G; rs156697) and other GSTs (GSTP1, GSTM1, GSTT1, GSTZ1) Main findings: - positive association between GSTO2*N142D variant and bladder cancer risk independently of arsenic exposure.	[71]
Muscle invasive bladder cancer	Serbia	105 patients	Polymorphisms: GSTO1*A140D (C419A; rs4925), GSTO2*N142D (A424G; rs156697), and other GSTs (GSTT1, GSTP1, GSTM1, GSTA1) Main findings: - worse prognosis and shorter survival for homozygous mutant GSTO1 Asp140Asp (*AA) and GSTO2 Asp142Asp (*GG) genotypes; - shorter survival in patients receiving chemotherapy and homozygous mutant for GSTO2 Asp142Asp.	[72]
Urothelial carcinoma of the bladder (UCB)	Taiwan	300 UC patients vs. 233 controls	Polymorphisms: GSTO1*A140D (C419A; rs4925), sulfotransferase 1A1 (SULT1A1)*R213H Main findings: - increased UC risk in subjects with GSTO1 Ala/Ala and SULT1A1 Arg/Arg variants; - increased UC risk in heavy smokers with GSTO1 Ala/Ala and SULT1A1 Arg/Arg variants; - synergistic effect between the GSTO1 Ala/Ala SULT1A1 Arg/Arg genotype on UCB risk.	[73]
Transitional cell carcinoma (TCC) of urinary bladder	Serbia	187 patients vs. 140 controls	Polymorphisms: GSTO1*A140D (C419A; rs4925), GSTO2*N142D (A424G; rs156697) Main findings: - increased TCC risk in subjects with homozygous mutant GSTO2 Asp142Asp genotype (*G/G); - combined effect of wild-type GSTO1 (*C/C or *C/A) genotypes and mutant GSTO2 (*G/G) on TCC risk (haplotype CG); - combined effect of mutant GSTO2 (*G/G) genotype and smoking on TCC risk.	[74]
Non-muscle invasive bladder cancer (NMIBC)	China	Discovery cohort 244 patients Validated cohort 86 HCC patients	Polymorphisms: GSTO1*A140D (C419A; rs4925), GSTO2* N142D (A424G; rs156697), GSTP1 (rs1695), ABCB1 (rs3747802, rs3213619) Main findings: - unfavorable recurrence-free survival (RFS), mean survival time (MST), and epirubicin-related toxicity in subjects with at least one GSTO1*A140D variant (AC and AA genotypes); - no correlations in mitomycin-treated subjects; - no effect related to GSTO2* N142D variants.	[75]
Non-muscle invasive bladder cancer (NMIBC)	USA	414 patients	Polymorphisms: A total of 114 SNPs of 21 genes involved in glutathione pathway including GSTO1 rs4925 (C419A), rs11509438 (G622A), GSTO2 rs641071 (-1102 T/G), rs156697 (A424G), and rs3740466 (3′UTR G/A) polymorphisms Main findings: - no significant associations of GSTO polymorphisms and cancer recurrence in patients who underwent to transurethral resection (TUR) alone or in combination with intravesical bacillus Calmette Guérin instillation (BCG) therapy.	[76]
Skin lesions	India	229 cases vs. 199 controls	Polymorphisms: GSTO1*A140D (C419A; rs4925), GSTO1*E208K (G622A; rs11509438), GSTO1*E155del (AGG deletion; rs11509437), GSTO2*N142D (A424G; rs156697), and polymorphisms of purine nucleoside phosphorylase (PNP), arsenic (+3) methyltransferase (As3MT). Main findings: - no association between GSTO1-1 or GSTO2-2 variants and arsenic-induced skin lesions.	[39]
Skin lesions	China	331 patients vs. 519 controls	Polymorphisms: GSTO1 SNPs: rs11191979, rs2164624 (-1242 G/A), rs2282326, rs4925 (C419A); GSTO2 SNPs: rs156697 (A424G), rs157077 (C20T), and rs2297235; other SNPs in PNP. Main findings: - increased risk of arsenic-related skin lesions in carriers of at least one GSTO1*C allele (rs11191979), GSTO1*A allele (rs2164624), and GSTO1*A allele (rs4925); - increased risk of arsenic-related skin lesions in carriers of at least one GSTO2*G allele (rs2297235); - increased risk of arsenic-related skin lesions in carriers of the AG genotype for GSTO2 rs156697 and rs2297235; - increased risk of arsenic-related skin lesions in carriers of haplotype CT between rs4925 and rs11191979 and haplotype GCG among rs156697, rs157077, and rs2297235.	[49]
Premalignant skin lesions	Bangladesh	594 patients vs. 1041 controls	Polymorphisms: GSTO1*A140D (C419A; rs4925), GSTO1*E208K (G622A; rs11509438), GSTO1*E155del (AGG deletion; rs11509437), and methylenetetrahydrofolate reductase (MTHFR rs1801133 and rs1801131) Main findings: - increased risk of skin lesions for subjects carrying the A/A genotype of GSTO1 C419A; - reduced risk of skin lesions for subjects carrying rs11509438 and rs11509437 variants of GSTO1-1; - elevated risk of skin lesions for subjects carrying the AAG/AAG diplotype for the three SNPs of GSTO1 analyzed.	[79]
Basal cell skin carcinoma (BCC)	Brazil	102 patients vs. 124 controls	Polymorphisms: GSTO2*N142D (A424G; rs156697) and variants or deletions of other GSTs (GSTM1, GSTT1, GSTP1) Main findings: - no significant difference between GSTO2-2 polymorphism levels in different types of skin tumors.	[80]
Hepatocellular carcinoma (HCC) cholangiocarcinoma colorectal cancer breast cancer	Thailand	28 cases of HCC, 30 cases of cholangiocarcinoma, 31 cases of colorectal cancer, 30 cases of breast cancer vs. 98 controls	Polymorphisms: GSTO1*A140D (C419A; rs4925), GSTO2*N142D (A424G; rs156697) Main findings: - association of GSTO1*A140D polymorphism with HCC, cholangiocarcinoma, and breast cancer; - no significant association of GSTO1*A140D polymorphism and colorectal cancer; - no significant association of GSTO2*N142D polymorphism with any type of cancer.	[38]
Hepatocellular carcinoma (HCC)	China	214 HCC patients	Polymorphisms: GSTO1: rs2282326; GSTO2: rs17116779, rs156699, rs7085725, rs157077 (C20T); other GSTs (GSTA1, GSTA4, GSTM2, GSTM3, and GSTP1) Main findings: - increased risk of death and a shorter median survival time in subjects carrying at least one variant allele of GSTO2 rs7085725; - increased risk of death in subjects with both GSTO2 rs7085725 and GSTP1 rs4147581 genotypes; - GSTO2 and GSTP1 gene polymorphisms may serve as independent prognostic markers for HCC.	[81]
Hepatocellular carcinoma (HCC)	China	228 patients	Polymorphisms: GSTO2: rs156699, rs157077 (C20T) and rs7085725 Main findings: - significant association between rs157077 variant of GSTO2-2 and OS of transarterial chemoembolization (TACE)-treated HCC patients.	[82]
Hepatocellular Carcinoma (HCC)	Egypt	320 patients vs. 150 healthy controls	Polymorphisms: GSTO1*A140D (C419A; rs4925), GSTO2* N142D (A424G; rs156697) Main findings: - increased risk of hepatitis B virus (HBV) disease progression in subjects with GSTO1*A140 wild-type allele and GSTO2*N142D variant allele.	[83]
Cholangiocarcinoma (CCA)	USA	Discovery cohort 370 patients vs. 740 healthy controls Validation cohort 212 patients vs. 424 healthy controls	Polymorphisms: GSTO1*A140D (C419A; rs4925) and SNPs in other inflammation or cancer- associated genes (COX-2, IL6, IL6R, IL6ST, SULF1, VEGFA, WRAP53, NKG2D) Main findings: - no association between allele frequency of GSTO1*A140D variant (and SNPs of the other genes selected) and CCA risk or survival.	[84]
Lung cancer	USA	620 cases of COPD (432 with lung cancer and 188 without) vs. 893 controls	Polymorphisms: Five *GSTO1* polymorphisms (rs11191975, rs1147611, rs11509438, rs2164624, rs628480) and six *GSTO2* polymorphisms (rs10491045, rs11191990, rs156699, rs157077, rs157080, rs568526) as a part of 470 SNPs in 52 genes involved in glutathione metabolism, DNA repair, and inflammatory response pathways Main findings: - significant association between GSTO2-2 gene and COPD with lung cancer.	[87]
Lung cancer	USA	973 patients with both small cell and non-small cell lung cancer, with platinum-based chemotherapy.	Polymorphisms: Six *GSTO1* (including rs4925 and rs2164624) polymorphisms, nine *GSTO2* polymorphisms (including rs156697), and other SNPs in glutathione-associated enzymes Main findings: - no association between GSTO1-1 or GSTO2-2 polymorphisms analyzed and lung cancer survival upon cisplatin chemotherapy.	[88]
Non-small cell lung cancer (NSCLC)	Turkey	172 patients vs. 214 healthy controls	Polymorphisms: GSTO1*A140D (C419A; rs4925) Main findings: - no significant association between GSTO1*A140D variant and risk of NSCLC.	[89]
Head and neck squamous cell carcinoma (HNSCC)	Thailand	300 patients vs. 299 controls	Polymorphisms: GSTO1*A140D (C419A; rs4925), GSTO2*N142D (A424G; rs156697) Main findings: - no significant differences in GSTO1 and GSTO2 genotypes between HNSCC and controls; - significant association between wild-type GSTO1*A140 and nodal metastasis and advanced pathological stage; - no significant association between GSTO2*N142D variant and clinico-pathological features.	[91]
Gastric cancer	Iran	67 patients vs. 134 controls	Polymorphisms: GSTO2*N142D (A424G; rs156697) and polymorphisms of other GSTs (GSTM1, GSTT1) Main findings: - no association between GSTO2*N142D variant and gastric cancer risk; - decreased risk of gastric cancer in subjects without history of cancer in their first-degree relatives and with homozygous GSTO2*N142D (D/D) genotype.	[92]
Colorectal cancer	Iran	63 patients vs. 126 healthy controls	Polymorphisms: GSTO2*N142D (A424G; rs 156697) Main findings: - no association between GSTO2*N142D variant and gastric cancer risk; - increased risk of gastric cancer in subjects with history of cancer in their first-degree relatives and with homozygous wild-type GSTO2*N142 (N/N) genotype.	[93]
Clear cell renal cell carcinoma (ccRCC)	Serbia	239 patients vs. 350 controls	Polymorphisms: GSTO1*A140D (C419A; rs4925), GSTO2*N142D (A424G; rs156697), GSTO2 polymorphism in 5′UTR gene region (A183G; rs2297235) Main findings: - increased ccRCC risk in subjects with combined homozygous GSTO1*A140D (D/D) and GSTO2*N142D (D/D) variants; - increased risk of ccRCC in subjects with GSTO1*A (rs4925), GSTO2*G (rs156697), and GSTO2*G (rs2297235) haplotype (H2) compared to haplotype including all three referent alleles (H1); - increased risk of ccRCC in smokers with homozygous GSTO2*N142D (rs156697) variant.	[94]
Clear cell renal cell carcinoma (ccRCC)	Serbia	228 patients	Polymorphisms: GSTO1*A140D (C419A; rs4925), GSTO2*N142D (A424G; rs156697), *GSTO2* polymorphism in 5′UTR gene region (A183G; rs2297235). Main findings: - shorter survival in male carriers of GSTO1*C/C wild-type genotype (GSTO1*A140) compared to the carriers of at least one variant allele; - increased risk of overall mortality among male ccRCC patients with GSTO1*C/C wild-type genotype.	[95]
Thyroid cancer	Brazil	145 patients with thyroid nodules (follicular carcinomas, papillary carcinomas, follicular adenoma, multinodular goiters) vs. 173 controls	Polymorphisms: GSTO1*A140D (C419A; rs4925) Main findings: - no association between GSTO1*A140D polymorphism and risk and clinico-pathological features of thyroid nodules.	[96]
Thyroid cancer	Brazil	248 patients with thyroid nodules (benign goiters, follicular adenomas, papillary carcinomas, follicular carcinomas) vs. 277 controls	Polymorphisms: GSTO1*A140D (C419A; rs4925), polymorphisms of other GSTs (GSTM1, GSTT1, GSTP1), CYP1A1, and codon 72 of *p53* Main findings: - no association between GSTO1*A140D polymorphism (alone or in combination with other polymorphisms) and clinico-pathological features of thyroid nodules.	[97]
Breast cancer	Germany	1348 patients	Polymorphisms: GSTO1*A140D (C419A; rs4925) and other polymorphisms in oxidative stress-related genes Main findings: - no significant association between GSTO1*A140D variant and overall mortality or possible differential effects by radiotherapy treatment.	[30]
Breast cancer	Pakistan	100 patients vs. 100 healthy controls	Polymorphisms: GSTO2*N142D (A424G; rs156697) and polymorphisms of other GSTs (GSTM1, GSTT1, GSTP1) Main findings: - increased risk of breast cancer in postmenopausal woman homozygous for GSTO2*N142D; - increased risk of breast cancer in premenopausal women with the combined presence of GSTP1 and GSTO2 polymorphisms.	[31]
Breast cancer	Denmark	396 patients vs. 396 matched controls	Polymorphisms: GSTO1*A140D (C419A; rs4925), GSTA1*A. and GSTA1*B alleles. Main findings: - increased risk of estrogen receptor-positive breast cancer for postmenopausal women carrying homozygous GSTO1*A140D variant, as compared with homozygous wild-type; - no clear association between the GSTA1 polymorphism and breast cancer among postmenopausal women.	[98]
Breast cancer	Thailand	101 patients vs. 151 controls	Polymorphisms: GSTO1*A140D (C419A; rs4925), GSTO2*N142D (A424G; rs156697) Main findings: - no association between GSTO1-1 and GSTO2-2 genotypes and the risk of breast cancer; - higher prevalence of homozygous wild-type GSTO1*A140 genotype is significantly correlated with advanced-stage breast cancer.	[99]
Breast cancer	Germany	1021 patients vs. 1015 controls	Polymorphisms: GSTO1*A140D (C419A; rs4925), GSTO1 polymorphism in 5′UTR gene region (G1242A; rs2164624), GSTO2*N142D (A424G; rs156697), *GSTO2* polymorphism in 5′UTR gene region (A183G; rs2297235), and polymorphisms of other GSTs (GSTA2, GSTM2, GSTZ) Main findings: - no association between GSTO polymorphisms and breast cancer risk; - no association between GSTO polymorphisms and histopathological tumor characteristics.	[100]
Breast cancer	Iran	181 patients vs. 181 controls	Polymorphisms: GSTO2*N142D (A424G; rs156697), polymorphisms of other GSTs (GSTM1, GSTT1), and XRCC1 Main findings: - no association between GSTO2*N142D polymorphism (allele frequencies and genotypes) and breast cancer risk.	[101]
Breast cancer	Iran	153 patients vs. 150 healthy controls	Polymorphisms: GSTO1*A140D (C419A; rs4925), GSTO2*N142D (A424G; rs156697) Main findings: - increased risk of breast cancer in subjects with A allele of GSTO1*A140D (C419A; rs4925) and G allele of GSTO2*N142D (A424G; rs156697); - increased risk of breast cancer in subjects with CA+AA genotype for GSTO1-1 and GG genotype for GSTO2-2.	[102]
Ovarian cancer	Brazil	69 patients vs. 222 healthy controls	Polymorphisms: GSTO2*N142D (A424G; rs156697), polymorphisms of other GSTs (GSTT1, GSTM1, GSTP1), and codon 72 of *p53* Main findings: - no association between GSTO2*N142D variant and the risk of ovarian cancer.	[104]
Ovarian cancer	Thailand	20 patients vs. 41 controls	Polymorphisms: GSTO2*N142D (A424G; rs156697) Main findings: - increased risk of ovarian cancer in subjects with GSTO2*N142D (A424G) polymorphism.	[105]
Cervical cancer (CC)	Iran	50 CC patients vs. 43 patients positive for HPV vs. 43 controls	Polymorphisms: GSTO1*A140D (C419A; rs4925), GSTO2*N142D (A424G; rs156697) Main findings: - reduced risk of human papillomavirus (HPV) 6, 16, 18, and 16/18 infections and CC in subjects with GSTO1*D140 variant (GSTO1*A allele; rs4925), heterozygous GSTO1*A140D (*C/*A genotype), and the combination of heterozygous GSTO1*A140D (140AD) and homozygous wild-type GSTO2*N142 (142NN); no association between GSTO2*N142D polymorphism and HPV infections and cervical cancer.	[107]
Ovarian cancer (OC)	Serbia	110 patients vs. 129 controls	Polymorphisms: GSTO1*A140D (C419A; rs4925), GSTO2*N142D (A424G; rs156697) Main findings: - increased OC risk in homozygous carriers of GSTO2*G (A424G) variant allele; - reduced OC risk in carriers of both GSTO1*A variant allele and GSTO2*A wild-type one (haplotype H4; *A*A).	[106]
Testicular germ cell tumor (GCT)	Serbia	88 patients vs. 96 controls	Polymorphisms: GSTO1*A140D (C419A; rs4925), GSTO2*N142D (A424G; rs156697), *GSTO2* polymorphism in 5′UTR gene region (A183G; rs2297235) Main findings: - increased testicular GCT risk in subjects with GSTO1*C/A*C/C genotypes (rs4925; A/D, D/D) and in GSTO2*A/G*G/G genotypes (rs2297235); - increased testicular GCT risk in carriers of combined GSTO2*A/G*G/G (rs156697; N/D, D/D) and GSTO2*A/G*G/G (rs2297235) genotypes.	[34]
Prostate cancer	Brazil	125 patients vs. 100 benign prostatic hyperplasia patients	Polymorphisms: GSTO1*A140D (C419A; rs4925), polymorphisms of other GSTs (GSTT1, GSTM1, GSTP1), and CYP1A1 Main findings: - no significant difference in the frequency of the GSTO1*A140D polymorphism between patients with benign prostatic hyperplasia and those with prostate cancer; - no association of GSTO1*A140D polymorphism with parameters of aggressiveness or response to radiotherapy in prostate cancer patients.	[110]
Prostate cancer (PC)	Serbia	237 patients vs. 236 controls	Polymorphisms: GSTO1*A140D (C419A; rs4925), GSTO2*N142D (A424G; rs156697), and polymorphisms of other GSTs (GSTM1, GSTT1, GSTP1) Main findings: - increased PC risk in subjects with homozygous GSTO1*A/A (rs4925; D/D) and GSTO2*G/G (rs156697; D/D) variant genotypes; - increased PC risk in carriers of both GSTO1*A and GSTO2*G variant alleles (haplotype H2).	[111]
Acute lymphoblastic leukemia (ALL)	Thailand	99 patients vs. 100 controls	Polymorphisms: GSTO1*A140D (C419A; rs4925), GSTO2*N142D (A424G; rs156697) Main findings: - increased ALL risk in with heterozygous GSTO1*A140D variant; - significant association between GSTO2*N142D polymorphism and high-risk group of ALL.	[40]
Pre-B acute lymphoblastic leukemia (ALL)	Iran	100 patients vs. 120 controls	Polymorphisms: GSTO1*A140D (C419A; rs4925), GSTO2*N142D (A424G; rs156697) Main findings: - no association between allele frequency or genotypes of GSTO1*A140D and GSTO2*N142G variants and Pre-B ALL risk; - significant reverse correlation with Pre-B ALL risk, for 140AA/142DD or 140DD/142NN combination genotypes.	[112]
B-acute lymphoblastic leukemia (B-ALL)	India	150 patients vs. 150 controls	Polymorphisms: GSTO1*A140D (C419A; rs4925), GSTO2*N142D (A424G; rs156697), and polymorphisms of other GSTs (GSTM1, GSTT1, GSTP1) Main findings: - no association between GSTO1*A140D (allele or genotype frequencies) and B-ALL risk; - increased cancer risk in subjects with variant G allele (D140) and for heterozygous GSTO2-AG (N/D) and homozygous GSTO2-GG (D/D) variant genotypes; - significant association between GSTO1*C/GSTO2*G haplotype and B-ALL risk; - significantly lower DFS in subjects with GSTO2*GG (D/D) genotype.	[113]
Mantle cell lymphoma (MCL)	USA	89 patients treated with R-HyperCVAD at ten-year follow-up	Polymorphisms: Three *GSTO1* polymorphisms (rs1147611, rs4925, rs2164624), three *GSTO2* polymorphisms (rs156697, rs157080, rs568526), and other polymorphisms in genes involved in GSH metabolism and DNA damage repair pathways Main findings: - significant associations between three *GSTO1* polymorphisms (rs1147611, rs4925, rs2164624) and OS; - significant association between two *GSTO2* polymorphisms (rs156697, rs157080) and OS.	[114]

## 5. Conclusions

The analysis of the data from the literature highlights some discrepancies about the significance of GSTO polymorphisms and cancer risk. However, it must be considered that certain limitations in the available studies hinder a definitive understanding of this matter, not helping clarify the true significance of these genetic variations.

A large number of studies focused on GSTO polymorphisms in bladder and breast cancers, followed by liver and skin malignancies. An uncritical evaluation of the data from the literature may show that some studies find an increased risk of a certain type of neoplasia in the presence of GSTO1*A140D or GSTO2*N142D polymorphisms, while just as many studies do not find significant correlations. A very limited number of studies have demonstrated the protective effects of major GSTO polymorphisms. Finally, only a small portion of studies investigated less frequent polymorphisms, such as GSTO1*E208K or GSTO1*E155del.

These studies present several limitations, including the following: (i) a predominant focus on the most frequent polymorphisms of GSTOs; (ii) challenges in comparing studies that analyze single polymorphisms with those analyzing even complex haplotypes; (iii) a limited number of investigations into the functional impact of GSTO polymorphisms on enzymatic functions, especially those affecting intracellular prosurvival signaling pathways, such as glutathionylation/deglutathionylation; (iv) a paucity of studies on some types of neoplasms, some of which are geographically restricted to a single region; and (v) a considerable variation in the sample size across the various studies (median (numbers of patients recruited): 187; range: 20–1348).

However, given the numerous variables involved, it would be misleading to draw a general model from the literature that is valid for all tumor types. The factors contributing to these discrepancies can be summarized as follows:

1. The physiological levels of GSTOs’ expression in the tissue/organ from which the tumor originates;
2. The presence of alternative mechanisms involved in GSTOs-catalyzed reactions (e.g., arsenic metabolism);
3. Different GSTOs’ polymorphism frequencies and types among ethnic groups;
4. The cancer type, sources of the control, and sample size, i.e., the number of patients recruited, to generate a valuable hypothesis;
5. Environmental factors. As stated above, GSTs are classified as low-penetrance genes that contribute to cancer risk when combined with environmental factors (e.g., arsenic, smoking, drugs, etc.);
6. The relevance of the specific “mosaic” of functional GSTOs’ polymorphism alterations for a specific tumor; e.g., MMA^V^ and DMA^V^ reductase variations may have consequences in the presence of suitable arsenic concentrations and in some specific tissues; GSTO substrates of glutathionylation/deglutathionylation (and their alterations) may vary in influencing the progression of a specific cancer.

The different and specific roles of GSTOs, especially in regulating intracellular pathways related to cell survival, proliferation, drug resistance, and inflammation through S-thiolation, suggest that for a better understanding of GSTOs’ polymorphisms, the cellular/tissue context (e.g., tumor type, stroma, etc.) and the impact of extrinsic (environmental) factors that GSTOs may directly or indirectly modulate should be taken into account. Such an approach is well-suited for investigations extended to haplotypes that include GSTO variants as well as other factors directly implicated in the pathogenesis of a specific type of neoplasm. Further research into haplotypes combining GSTOs variants and polymorphisms in other factors more specifically implicated in the pathogenesis of a specific cancer type would support this goal. Finally, expanding studies on tumor types that have been minimally or not yet investigated in relation to GSTO polymorphisms could help to define a better picture of their role in cancer.

## Figures and Tables

**Table 1:** Comparison of specific activities of GSTO1-1 and GSTO2-2.

Enzymatic Activity	GSTO1-1	GSTO2-2
DHA reductase	Yes [12,13]	Yes [8,12]
Inorganic arsenate reductase	Yes [15]	-----
MMA^V^ reductase	Yes [12,14]	Yes [12]
DMA^V^ reductase	Yes [12,14]	Yes [8,12]
S-(Phenacyl)glutathione reductase	Yes [17]	No [17]
Glutaredoxin (Grx)-like thioltransferase reactions	Yes [5,16]	Yes [6,12]
Deglutathionylation	Yes [6]	No [6]
Glutathionylation	Yes [18]	Yes [18]
Classical glutathione transferase	Yes [5]	Yes [8]; No [12]

**Table 2:** Functional effects of GSTO1-1 and GSTO2-2 main polymorphisms.

Enzymatic Activity	GSTO1-1 A140D	GSTO1-1 E155del	GSTO1-1 E208K	GSTO2-2 N142D
DHA reductase	NC [7,12]	↑↑ [12]	NC [12,18]	NC [12]
MMA^V^ reductase	NC [7,12]	↑↑ [12]	NC [7] ↑ [12]	NC [7,16]
DMA^V^ reductase	NC [18]	↑↑ [12]	↑↑ [12]	NC [12]
S-(phenacyl)glutathione reductase	NC [18]	NC [17]	NC [17]	-----
Glutaredoxin (Grx)-like thioltransferase reactions	NC [7,12] ↓ [44]	↑↑ [7,12]	↑ [12] NC [18]	NC [12,16]
Deglutathionylation	↓ [18]	-----	↓ [18]	-----
Glutathionylation	↑↑ [18]	-----	-----	-----
Classical glutathione transferase	NC [18,44]	↑↑ [7]	-----	-----

NC: no change; ↑↑: increased; ↑ = slightly increased; and ↓ = decreased.
